# High prevalence of orocutaneous features in Yemeni patients with tuberous sclerosis complex

**DOI:** 10.1016/j.jdin.2025.11.013

**Published:** 2025-11-29

**Authors:** Mohammad Ali Alshami, Ahlam Mohamed Al-shami, Mona Jameel Mohana, Amat Alkhaliq Mohammed Al-Sayaghi, Hadeel Mohammad Alshami

**Affiliations:** a Dermatology Department, Faculty of Medicine and Health Sciences, Sana'a University, Sana'a, Yemen; b Department of Conservative Dentistry, Faculty of Dentistry, Sana'a University, Sana'a, Yemen

**Keywords:** angiofibroma, ash leaf, shagreen plaque, tuberous sclerosis complex, ungual fibroma, Yemen

**Figure:**
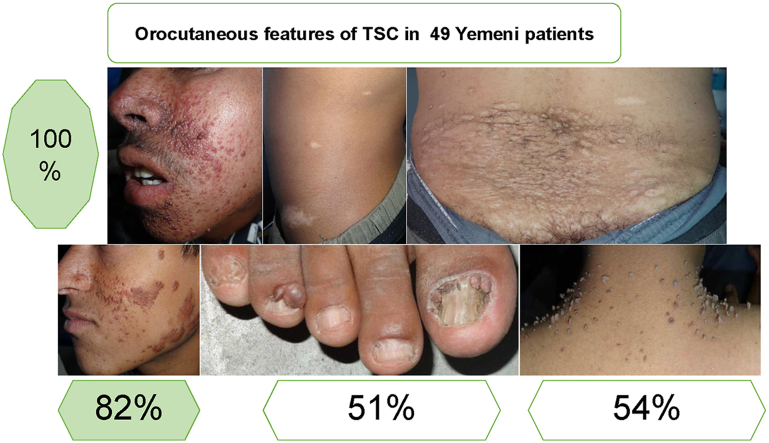

*To the Editor:* Tuberous sclerosis complex (TSC), an autosomal-dominant disorder caused by pathogenic variants in the *TSC1* or *TSC2* tumor suppressor genes. These mutations lead to unchecked cellular proliferation and hamartoma development in multiple organs, most notably the brain, kidneys, and skin. Orocutaneous features are among the earliest and most visible manifestations of TSC and remain critical diagnostic indicators, particularly when access to genetic testing is limited.^1^, ^2^, ^3^

We retrospectively reviewed 180,000 medical records at a dermatology referral center in Yemen spanning 25 years. In total, 49 patients (26 male; aged 2-42 years) met the 2012 International TSC Consensus Conference criteria for a definitive clinical diagnosis.^2^ All patients or their guardians provided written informed consent for study participation and image publication.

All patients presented with orocutaneous findings. Facial angiofibromas, hypomelanotic macules, and shagreen plaques were universally observed. Fibrous cephalic plaques (FCPs) were present in 40 (82%) patients, ungual fibromas in 25 (51%), fibroepithelial polyp in 22 (45%), and confetti-like hypopigmentation in 3 (6%). Oral examinations, performed in 17 patients, revealed oral fibromas in 15 (88%) and dental enamel pits in 12 (71%) patients. Six (12%) patients had a positive family history of TSC; the remainder were presumed to have sporadic mutations.^4^

Table I presents the frequency of cutaneous findings in TSC, while Supplementary Figs 1 to 7, available via Mendeley at https://data.mendeley.com/datasets/483tvs643z/1 illustrate their features. The prominence of facial angiofibromas (Fig 1, Supplementary Fig 1, available via Mendeley at https://data.mendeley.com/datasets/483tvs643z/1), hypomelanotic macules (Supplementary Fig 2, available via Mendeley at https://data.mendeley.com/datasets/483tvs643z/1), connective tissue nevi (shagreen plaques; Supplementary Fig 3, available via Mendeley at https://data.mendeley.com/datasets/483tvs643z/1)—so termed because they are elevated, contrasting patches that reflect only color changes; hence the term “shagreen patch” should be avoided and replaced by the correct terminology “plaque”—FCPs (Supplementary Fig 4, available via Mendeley at https://data.mendeley.com/datasets/483tvs643z/1), and ungual fibromas (Supplementary Fig 5, available via Mendeley at https://data.mendeley.com/datasets/483tvs643z/1) underscores their importance as diagnostic hallmarks.^5^ FCPs and fibroepithelial polyp (Supplementary Fig 6, available via Mendeley at https://data.mendeley.com/datasets/483tvs643z/1)—clinically similar to skin tags, while larger, more numerous, and with an earlier onset—were especially common, contrasting earlier reports suggesting lower prevalence.^5^ Similarly, oral fibromas (Supplementary Fig 7, available via Mendeley at https://data.mendeley.com/datasets/483tvs643z/1) and DEPs, although often under-recognized, were frequently detected under examination, emphasizing the need for multidisciplinary evaluation.

**Table I:** Frequency of orocutaneous and oral features in Yemeni patients with tuberous sclerosis complex (*N* = 49)

Feature	Frequency (%)
Facial angiofibromas (*n* = 49)	49 (100)
Hypomelanotic macule (*n* = 49)	49 (100)
Shagreen plaque (*n* = 49)	49 (100)
Fibrous cephalic plaque (*n* = 49)	40 (82)
Ungual fibroma (*n* = 49)	25 (51)
Molluscum fibrosum pendulum (*n* = 49)	22 (45)
Confetti-like hypopigmentation (*n* = 49)	3 (6)
Oral fibroma (*n* = 17)	*15 (88)*
Dental enamel pit (*n* = 17)	*12 (71)*
Family history (*n* = 17)	*6 (12)*

Listed in descending order of frequency; oral features are shown in italics.

**Fig 1:**
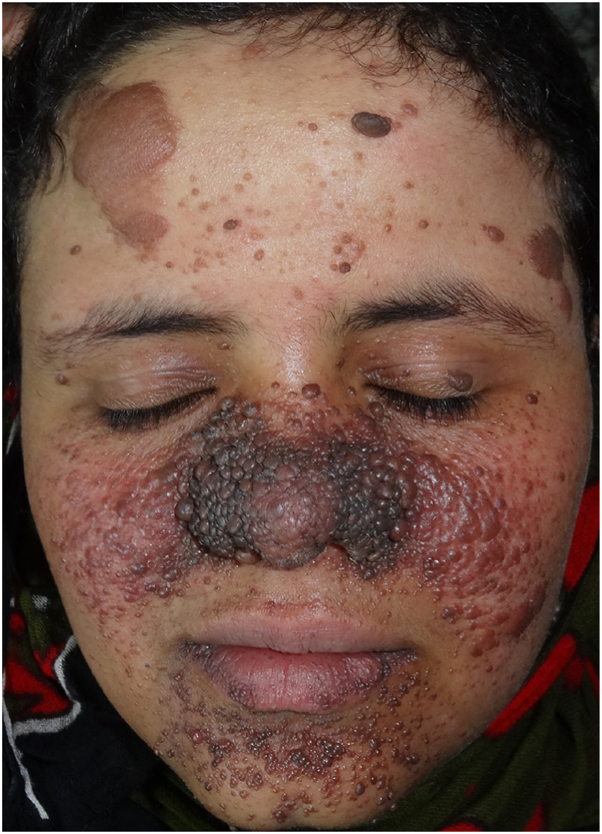
Extensive facial angiofibromas distributed bilaterally and symmetrically over the nasolabial areas and chin, along with multiple cephalic fibrous plaques on the forehead and left temple in a 21-year-old female patient. *TSC*, Tuberous sclerosis complex.

FAFs should not be confused with similar lesions, such as acne, trichoepitheliomas, or syringomas, as they present earlier and are confined to the centrofacial region. Hypomelanotic macules are differentiated from other hypopigmented lesions by their characteristic lanceolate or confetti-like shapes. Fibroepithelial polyp can be misdiagnosed as skin tags; their larger size, greater number, earlier onset, and association with other TSC features support accurate diagnosis. Shagreen plaques may resemble nevus lipomatosus superficialis because of their soft consistency, irregular surface, and distribution. Although FCPs can mimic other elevated lesions of the face and scalp, their characteristic color, firm consistency, head and neck limited distribution, and association with other TSC features are critical in diagnosis confirmation. Finally, ungual fibromass should be distinguished from acquired ungual fibromas.

Although this study is limited by its retrospective design and incomplete oral assessments, the consistency and visibility of orocutaneous features support their utility in early clinical suspicion and diagnosis of TSC. These findings are relevant for dermatologists and dental professionals serving in resource-limited settings.

## Conflicts of interest

None disclosed.
